# Antifungal compounds, GC-MS analysis and toxicity assessment of methanolic extracts of *Trichoderma* species in an animal model

**DOI:** 10.1371/journal.pone.0274062

**Published:** 2022-09-23

**Authors:** Afrasa Mulatu, Negussie Megersa, Teshome Tolcha, Tesfaye Alemu, Ramesh R. Vetukuri

**Affiliations:** 1 Department of Microbial, Cellular and Molecular Biology, Addis Ababa University, Addis Ababa, Ethiopia; 2 Department of Chemistry, Addis Ababa University, Addis Ababa, Ethiopia; 3 Department of Chemistry, Kotebe University of Education, Addis Ababa, Ethiopia; 4 Department of Plant Breeding, Swedish University of Agricultural Sciences, Alnarp, Sweden; Tocklai Tea Research Institute, INDIA

## Abstract

Fungi of the genus *Trichoderma* have been marketed for the management of diseases of crops. However, some *Trichoderma* species may produce toxic secondary metabolites and it should receive due attention to ensure human safety. In this study, we investigated the *in vitro* antagonistic potential of *T*. *asperellum* AU131 and *T*. *longibrachiatum* AU158 as microbial biocontrol agents (MBCAs) against *Fusarium xylarioides* and the associated antagonistic mechanism with bioactive substances. Swiss albino mice were used to evaluate the *in vivo* toxicity and pathogenicity of *T*. *asperellum* AU131 and *T*. *longibrachiatum* AU158 methanolic extracts and spore suspensions, respectively, in a preliminary safety assessment for use as biofungicides. Gas Chromatography-Mass Spectrometry (GC-MS) was used to profile volatile organic metabolites (VOCs) present in the methanolic extracts. The agar diffusion assay of the methanolic extracts from both *T*. *asperellum* AU131 and *T*. *longibrachiatum* AU158 were effective at a concentration of 200 μg/mL (1×10^7^ spores/mL), causing 62.5%, and 74.3% inhibition, respectively. A GC-MS analysis of methanolic extracts from both bioagents identified 23 VOCs which classified as alcohols, acids, sesquiterpenes, ketones and aromatic compounds. The oral administration of methanolic extracts and spore suspensions of each *Trichoderma* species to female Swiss albino mice over 14 days did not show any significant signs of toxicity, mortality or changes to body weight. It can be concluded that the tested spore suspensions and methanolic extracts were not pathogenic or toxic, respectively, when administered to Swiss albino mice at various doses.

## Introduction

Given the large socio-economic impact of crop monocultures and the environmental hazards of chemical pesticides [1], microbial biocontrol agents (MBCAs) have recently become a strategic option for controlling plant diseases [2], insects [3] and weeds [4]. A number of biopesticides that contain bioagents such as *T*. *asperellum*, *T*. *harzianum* and *T*. *viride* as the active ingredients are currently marketed in Europe, USA, Asia and Africa [5, 6]. In contrast to chemical pesticides, which have been reported to induce resistance among pests and cause residual toxic effects, biopesticides are highly selective against the target pest [6]. Microbial biocontrol agents (MBCAs) have been designed to grow and reproduce, survive in the environment for prolonged periods in symbiotic consortia with in the host. Hence, a relatively small amount of MBCAs are needed to be applied to a certain location compared to chemical pesticides [5, 6]. Although the vast majority of MBCAs are generally regarded as safe for humans and the environment [7, 8], some studies have demonstrated that increased exposure to fungal substances among agricultural workers may affect the immune system [13, 14].

There are evidences that members of the genus *Trichoderma* are effective MBCAs against various plant pathogens across different agro-ecological systems [6, 9–12]. However, *Trichoderma* species are also known to cause opportunistic infections in humans, varying from localized to fatal disseminated diseases; these are particularly dangerous for risk populations, including patients undergoing peritoneal dialysis, transplant recipients and patients with hematological malignancies [13, 14]. In one of our previous studies, it has been reported that a comparatively high *Trichoderma* species diversity in coffee ecosystem in Ethiopia among which *T*. *asperellum* AU131 and *T*. *longibrachiatum* AU158 were found effective against *F*. *xylarioides* causing coffee wilt disease (CWD) [15]. Based on subsequent bioassay analysis under different conditions, we formulated a biofungicide from *T*. *asperellum* AU131 and *T*. *longibrachiatum* AU158 for the control of CWD caused by *F*. *xylarioides* [16]. However, the toxicological assessment of these bioagents were not conducted. Thus, systematic study is very important to evaluate and ensure the safety of biofungicides for agricultural use [17]. On the other hand, understanding and evaluating the mechanism of action of secondary metabolites of these potential MBCAs against *F*. *xylarioides* is very important. Several *Trichoderma* strains were widely studied due to their capacity to compete for nutrients and space [18], parasitize other fungi [19, 20], enact antibiosis by producing secondary metabolites or antimicrobial compounds [8, 21, 22], induce defense responses in plants [23], and to promote plant growth [24, 25]. Some strains of *T*. *longibrachiatum* and *T*. *orientale* are found to be toxic to humans, especially in immunocompromised patients [13, 14].

Since the 1960s, toxicity studies have been used as a vital step in the approval of new products onto the market. The present study was conducted in animal models following the protocols laid out by the Organization for Economic Cooperation and Development (OECD) [26]. More specifically, the study applied the experimental design described in Tier 1 of the Toxicological Evaluation of Microbial Pest Control Agents (MPCA), which has the purpose of assessing the pathogenicity and toxicity of biopesticide products [27]. In Europe, Council Directive 91/414/EEC and its successive amendments identify which requirements an applicant must achieve for the authorization to produce and market pesticides, including those that have a bioactive substances [28, 29]. In particular, the directive requires the provision of information concerning the short-term toxicity of any relevant metabolites produced by the candidate MBCA [29–31].

The present study provides toxicological data that are of importance in assessing the toxicological relevance of two potential MBCAs, *T*. *asperellum* AU131 and *T*. *longibrachiatum* AU158. To this end, it is conceivable that it would be more effective to determine the toxicological risks associated with a particular MBCAs by assaying a mixture of metabolites, like those in methanolic extracts, on model animals that are sensitive to a large spectrum of molecules, instead of assessing the toxicity of single and pure metabolites [28, 29]. Moreover, secondary metabolites produced by *Trichoderma* species should be identified and profiled, which is very helpful to categorize them according to their toxicological effects. Thus, the volatile metabolites were analysed by gas chromatography with a single quadrupole mass spectrometer detector (GC-MS) analysis [32, 33].

In the present study, Swiss albino mice were used to assess the acute toxicity of spore suspensions and methanolic extracts of *T*. *asperellum* AU131 and *T*. *longibrachiatum* AU158 in short term assays. Thus, the study aimed to: (i) evaluate the *in vitro* antagonistic activity of methanolic extracts against *F*. *xylarioides* (ii) evaluate the sensitivity of albino mice to spore suspensions and methanolic extracts; (iii) generate toxicological data that can be used when assessing the risk associated with the toxicity *Trichoderma* species; and (iv) profile volatile secondary metabolites produced by these fungi through GC-MS analysis.

## Materials and methods

### *Trichoderma* species and preparation of spore suspensions

When studying acute oral toxicity, spore suspensions (live cells) were prepared by adding 5 mL (0.9%) of NaCl to mature *Trichoderma* species on PDA plates to dislodge the spores from the mycelium [34]. The spore density was counted using a haemocytometer (Neuberger GmbH, Germany) to determine the spore concentrations.

### Effect of temperature and pH on mycelial growth of *Trichoderma* species

To evaluate the virulence of the MBCAs, mycelial discs of test fungus at different temperatures, from 25 to 37°C, were inoculated onto Petri dishes containing minimal (0.5% glucose, 0.1% KH_2_PO_4_, 0.1% (NH_4_)_2_SO_4_, 0.1% MgSO_4_.7H_2_O, 2% agar in distilled water) or yeast extract (0.5% glucose, 0.2% yeast extract, 1% KH_2_PO_4_, 2% agar in distilled water) agar medium. The effect of pH on mycelial growth was determined by using buffer solutions to adjust the pH of both minimal and yeast extract agar medium to values ranging from 2 to 9 [13]. Mycelial discs (5 mm in diameter) cut from the active growing culture were used as inocula. After incubation of inoculated plates in completely randomised desgin (CRD) at different temperatures, mycelial growth was determined by colony diameter measurements.

### Extraction of secondary metabolites

For secondary metabolite extraction, *Trichoderma* species were grown in 500 mL flasks containing 100 g (dry weight) solid substrate (wheat bran to white rice (2:1 *w/w*)) supplemented with 1% (*v/v*) glycerol (98.9%) and 1% (*w/v*) (NH_4_)_2_SO_4_) (99.5%) to moisten the substrate [16]. The flasks were sterilized at 121°C for 15 min and, after cooling, inoculated with 20 mL spore suspension (1 × 10^7^ spores/mL) followed by incubation at 28°C for 21 days [16]. The cultures were homogenized by adding methanol, followed by centrifugation at 5000 rpm for 15 min. The supernatant was collected and dried under a vacuum; the obtained methanolic extracts was purified using Sephadex LH-20 column (Sigma-Aldrich, St. Louis, MO) and stored at -20°C until use for oral administration in mice and GC-MS analysis.

### Toxicological bioassay of secondary metabolites against *F*. *xylarioides*

#### Seeded agar assay

The methanolic extracts of *T*. *asperellum* AU131 and *T*. *longibrachiatum* AU158 were filtered through 0.22 μm nylon membranes to obtain purified extracts. The methanolic extracts were mixed with warm SFM agar (200, 100, 50 and 10 μg/mL of methanolic extracts) and plated in sterile Petri plate to analyze the effect of non-volatile metabolites on the test pathogen [35]. A 5 mm mycelial disc of the test pathogen was placed at the center of the Petri plate-containing SFM agar. Plates without methanolic extracts was served as control. These plates were maintained at 28°C until the control plate was fully-grown. The diameter (mm) of the fungal colony growth was measured, and the percent of growth inhibition (PGI) was calculated using the formula,

$$PGI=\frac{C-T}{C}\times 100$$

where, *C* = radial growth of the pathogen in control plate; *T* = radial growth of the pathogen in treatment.

#### Agar diffusion assays

To evaluate antifungal activity of the methanolic extracts, 1 mL of spore suspension (5 × 10^5^ spore/mL) of *F*. *xylarioides*, was spread on the surface of the plate containing King B (KB) medium [36, 37]. Holes (5 mm diameter) were made in the center of the agar plates into which the test solutions or solvent control (MeOH) were applied [36, 37]. Fifty μL of the series dilution of the methanolic extracts were poured into the well and methanol was used as negative control. The plates were incubated in CRD at 25°C for 3 days. All bioassays were performed in triplicate and compared with identically prepared solvent controls. The inhibition zones were measured as the diameter of inhibition zones (clear zones) and expressed as the PGI. The size of the *F*. *xylarioides* growth inhibition zone was calculated in mm. The plates were photographed with a Canon digital camera and analyzed by using the graphic software “Fiji ImageJ” (GNU General Public License). Finally, the PIG was calculated using the following formula [38].

$$PIG=\frac{\left[(T-I)-(C-I)\right]}{\left(T-I\right)}\times 100$$

Where *T* = diameter of inhibition zones in in the treatment; *I* = initial hole diameter (5 mm) and *C* = diameter of inhibition of the solvent control.

### GC-MS metabolite profiling of *Trichoderma* species methanolic extracts

The volatile metabolites were analysed by gas chromatography with a single quadrupole mass spectrometer detector (GC-MS) analysis [32, 33]. Prior to the analysis, dried methanolic extracts obtained from *Trichoderma* species were dissolved in ethyl acetate and placed in a 1 mL glass vial. The GC system was equipped with an HP-5MS (30 m × 0.25 mm and 0.25 μm 5% diphenyl/95% dimethylpolysiloxane) capillary column (J and W Scientific, Folsom, CA, USA). The instrument was programmed to start at 40°C (held for 2 min) and to increase to 160°C by 6°C /min, after which it increased to 260°C by 10°C/min (held for 4 min). Helium (99.9%) was used as the carrier gas, and the flow rate was 1 mL/min. The ion source temperature was set at 230°C, the ionizing electron energy was 70 eV, and the mass range was 40–450 Da in full-scan acquisition mode [37, 38]. The spectra of the identified volatile metabolites were compared with the spectra of known compounds in the GC-MS of National Institute of Standards and Technology (NIST) database. The threshold for identification was ≥ 90% similarity. The analysis was carried out in triplicate to monitor the repeatability of the analysis. The instrumental responses obtained were interpreted using mass hunter ChemStation software.

### Experimental animal and ethical approval

Adult female Swiss albino mice (6–8 weeks old), nulliparous, non-pregnant and weighing 25–30 g [31, 39], were obtained from the Ethiopian Public Health Institute (Addis Ababa, Ethiopia). Female mice were allocated in treatment groups (five animals/per group). All of the animals were kept in polypropylene cages and acclimatized for a period of seven days prior to the start of the experiment. They were placed under standard conditions (23 ± 2°C with a 12 h light-dark cycle), and were fed commercial rodent chow *ad libitum* and given clean tap water during the experimental period [17]. All of the animals were cared for in compliance with the internationally accepted guide for the care and use of laboratory animals [40], as well as the Addis Ababa University institutional guidelines for animal ethics, which were previously approved by the College of Natural and Computational Sciences Institutional Review Board (CNCS-IRB) under the protocol number IRB/44/2020.

### Experimental design

For the effective administration of methanolic extracts and spore suspensions to mice through oral gavage, water was not provided in the morning to induce thirst. After fasting for 3 h, methanolic extracts and live cells (spore suspensions) were administered to each treatment group [31]. Prior to the administration of methanolic extracts and spore suspensions, the body weights of the mice were measured using analytical balance to prepare appropriate doses [31, 41, 42]. The animals in the experimental groups (n = 5) were administered with four distinct concentrations of spores (1 × 10^6^, 1 × 10^7^, 1 × 10^8^ and 1 × 10^9^ spores/mL) and four distinct concentrations of methanolic extracts (600, 1200, 2000 and 5000 mg/kg bw). A total of five female mice received each of the tested doses. Mice in the control group received the same volume of 0.9% NaCl [43].

### Acute oral toxicity

Single-dose acute oral toxicity was evaluated based on OECD Guidelines [39, 44]. The general behavior of mice and signs of toxicity (hypo-activity, breathing difficulty, tremors, and convulsion) were continuously observed for 1 h after oral treatment, after which these signs were intermittently observed for 4 h as well as over a period of 24 h [17, 39]. Attention was given to signs of tremors, convulsions, salivation, diarrhea, lethargy, sleep and coma. During the subsequent post-dosing period (14 days), the animals were observed at least once per day. All of the observations were systematically recorded, with individual records maintained for each animal. Body weights were measured at the initiation of treatment, as well as 7 and 14 days after administration [40]. The LD_50_ value was determined according to the Dragstedt and Lang method described by El Allaoui, Filali [45].

### Statistical analysis

Prism software (version 8.0; GraphPad, Inc., San Diego, CA) was used to perform the statistical analysis. Comparisons between the control and treatment groups were conducted using one-way analysis of variance (ANOVA), followed by a Tukey post-hoc test. All of the results from experiments performed in triplicates were presented as the mean value ± standard error of the mean; the threshold for statistical significance was set at p ≤ 0.05.

## Results

### Effect of temperature and pH on mycelial growth of *Trichoderma* species

**Fig 1:**
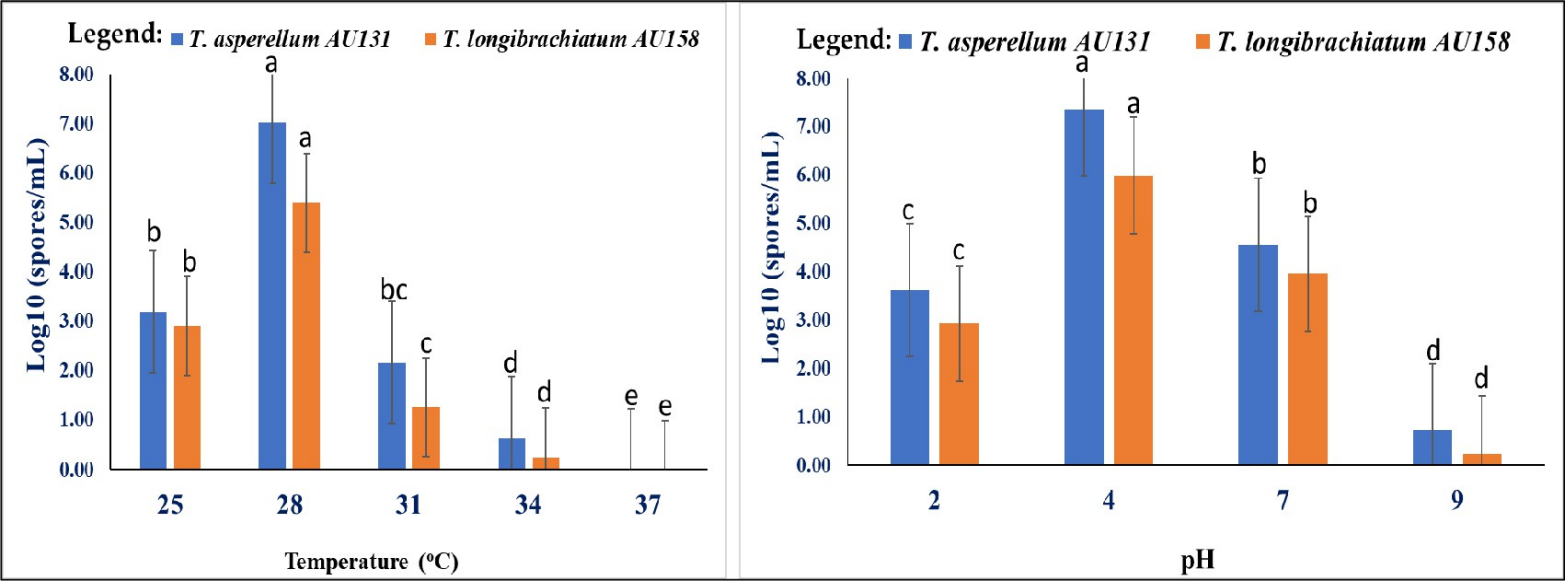
Effect of temperature and pH on *Trichoderma* species growth. Different alphabets depicted in superscript indicate mean treatments that are significantly different according to Tukey’s HSD posthoc test at p ≤ 0.05 each value is an average of 3 replicate samples ± standard error.

The investigated *Trichoderma* species showed optimum growth at 28°C on both minimal and yeast extract agar media, with neither species growing at 37°C (Fig 1). They were able to grow at all of the tested pH values (ranging from 2 to 9) at 28°C, with optimum growth observed at pH 4 (Fig 1). It should be stated that both MBCAs were able to grow at pH 7, which is a precondition of growth within the human body.

### Toxic effects of secondary metabolites against *F*. *xylarioides*

**Fig 2:**
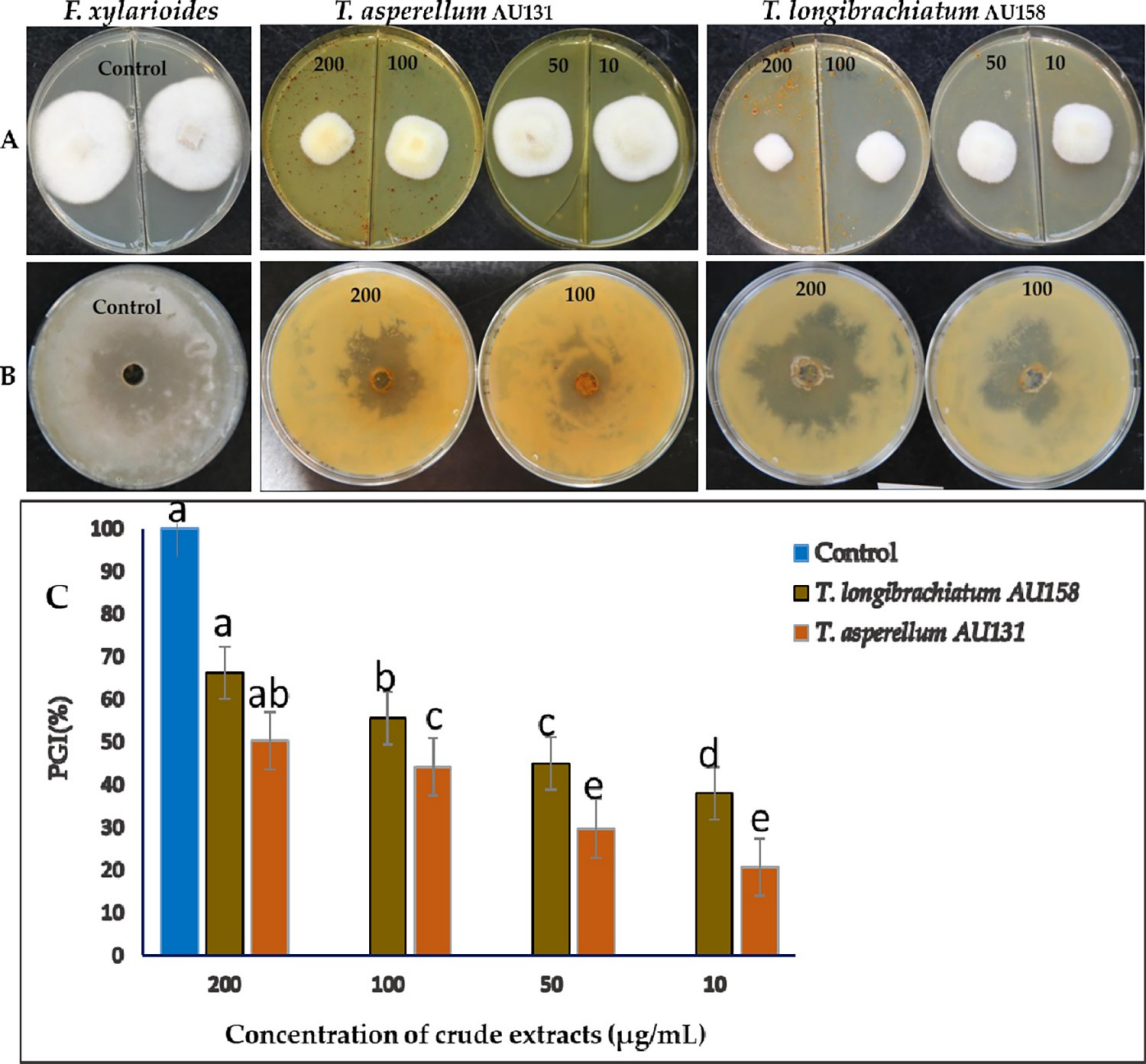
Bioassay activity of methanolic crude extracts of *T*. *asperellum* AU131 and *T*. *longibrachiatum* AU158 against *F*. *xylarioides*. **A** and **C:** Seeded agar assays: The picture shows the antifungal activity at different concentrations (200, 100, 50 and 10 μg/mL of crude extracts amended with PDA medium) and **B**: Agar diffusion assays (200 and 100 μg/mL of crude extracts). Different alphabets depicted in superscript indicate mean treatments that are significantly different according to Tukey’s HSD posthoc test at p ≤ 0.05, each value is an average of 3 replicate samples ± standard error. PGI = percentage of growth inhibition.

Methanolic extracts from both *Trichoderma* species amended with PDA medium were capable of inhibiting the mycelial growth of *F*. *xylarioides* (*p* ≤ 0.05). *Trichoderma longibrachiatum* AU158 showed the highest defined level of *in vitro* antagonistic activity in both seeded agar assay and agar diffusion assay methods (Fig 2A–2C). ANOVA analysis from seeded agar assay method revealed statistically significant (*p* ≤ 0.05) differences in the mycelial growth inhibition of *F*. *xylarioides* at different methanolic extracts concentrations, with inhibition percentages ranging from 38 to 66.2% (*T*. *longibrachiatum* AU158) and 20 to 50.2% (*T*. *asperellum* AU131) (Fig 2C). Moreover, the methanolic extracts from *T*. *longibrachiatum* AU158 at maximum concentrations incited a change in the colony morphology of the pathogen with significant reduction in growth. The mean inhibitory effect against *F*. *xylarioides* restricted almost completely in plates as compared to the control, *F*. *xylarioides*, grown alone (Fig 2A). The agar diffusion test of the methanolic extracts from both *T*. *asperellum* AU131 and *T*. *longibrachiatum* AU158 were the most effective at a concentration of 200 μg/mL, causing 62.5%, and 74.3% inhibition, respectively (Fig 2B). The inhibition was not visible on the test pathogen at a concentration ≤ 50 mg/mL. The radial growth inhibition of *F*. *xylarioides* in both bioassay methods are attributed to inhibitory secondary metabolites released by bioagents through competition, mycoparasitism and production of cell wall degrading enzymes.

### GC-MS identification of volatile metabolites

**Fig 3:**
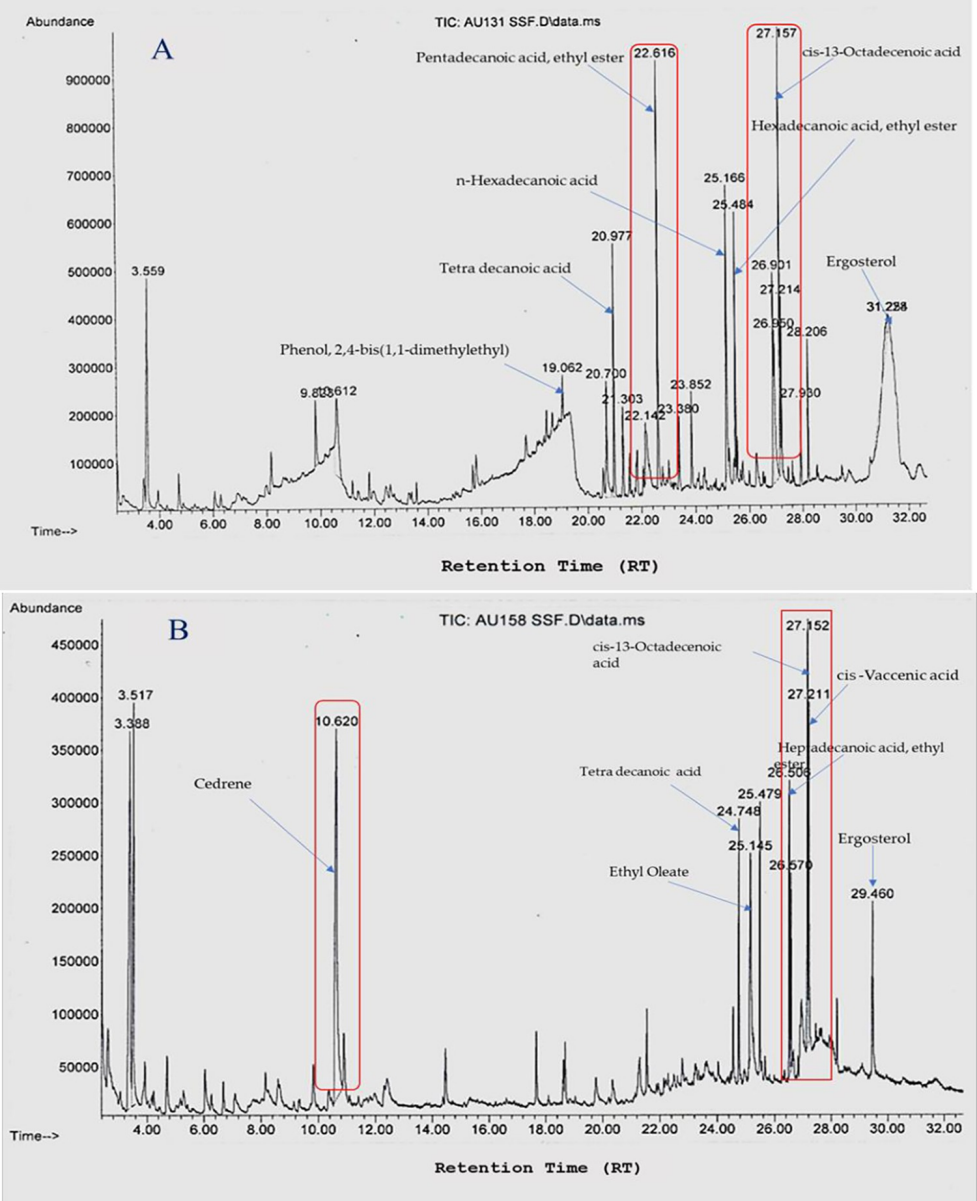
Chromatograms of the volatile organic compounds identified from *T*. *asperellum* AU131 (**A**) and *T*. *longibrachiatum* AU158 (**B**) crude extracts.

A GC-MS analysis of the methanolic extracts of both BCAs revealed the presence of 23 volatile secondary metabolites. The chromatogram illustrated that various volatile metabolites were present in the analyte, with these compounds then identified based on molecular weight, retention time, and molecular formula. Typical chromatograms and mass spectra of the compounds identified from the methanolic extracts of *T*. *asperellum* AU131 and *T*. *longibrachiatum* AU158 were shown in Fig 3A and 3B, respectively. The identified compounds were classified as alcohols, acids, sesquiterpenes, aromatics, ketones and esters according to the chemical class (Table 1). The most commonly identified VOCs from the *T*. *asperellum* AU131 methanolic extracts were; cis-13-Octadecenoic acid (21.48%), 2,4-bis(1,1-dimethylethyl)-Phenol (17.34%), *n*-hexadecanoic acid (15.6%), hexadecanoic acid ethyl ester (14.14%), and linoleic acid ethyl ester (11.46%), whereas cis-13-Octadecenoic acid (23.48%), 2,4-bis(1,1-dimethylethyl)-Phenol (18.5%), ethyl oleate (17.83%) and *n*-hexadecanoic acid (16.72) were detected from the *T*. *longibrachiatum* AU158 methanolic extracts (Table 1).

**Table 1:** Volatile organic compounds of *T*. *asperellum* AU131 and *T*. *longibrachiatum* AU158 identified by GC-MS.

Secondary Metabolites	*T*. *asperellum* AU131		*T*. *longibrachiatum* AU158		Chemical class	Molecular formula	Molecular weight (g/mol)
	RT (min)	Area (%)	RT (min)	Area (%)			
Benzene, 4-ethenyl-1,2-dimethoxy	15.68 ± 0.2	0.23 ± 0.1	ND		Aromatic	C_10_H_12_O_2_	164.20
2-(4-Methoxyphenyl) ethanol	15.81 ± 0.4	0.44 ± 0.3	ND		Alcohol	C_9_H_12_O_2_	152.19
Cedrene	ND		10.62 ± 0.5	0.16 ± 0.1	Sesquiterpenes	C_15_H_24_	204.19
2,4-bis(1,1-dimethylethyl)-Phenol	19.06 ± 1.3	17.34 ± 0.5	19.35 ± 0.4	18.5 ± 0.5	Sesquiterpenes	C_14_H_28_O	206.17
Tetradecanoic acid	20.98 ± 1.1	0.60 ± 0.5	24.75 ± 0.9	0.75 ± 0.9	Acid	C_14_H_22_O_2_	228.38
Pentadecanoic acid, ethyl ester	22.62 ± 0.9	0.16 ± 0.3	24.76 ± 1.1	0.38 ± 0.1	Esters	C_17_H_34_O_2_	270.46
Ethyl 13-methyl-tetradecanoate	23.38 ± 2.1	1.05 ± 0.4	24.8 ± 0.7	0.4 ± 0.2	Esters	C_17_H_34_O_2_	270.46
Palmitoleic acid	ND		24.97 ± 2.2	3.4 ± 0.2	Acid	C_16_H_30_O_2_	254.41
*n*-Hexadecanoic acid	25.17 ± 0.5	15.60 ± 0.7	25.48 ± 0.4	16.72 ± 0.7	Acid	C_16_H_32_O_2_	256.43
Hexadecanoic acid, ethyl ester	25.48 ± 1.6	14.14 ± 0.4	25.58 ± 1.6	11.84 ± 1.3	Ester	C_18_H_36_O_2_	284.48
Heptadecanoic acid	ND		26.22 ± 0.5	0.1 ± 0.2	Acid	C_17_H_34_O_2_	270.46
Heptadecanoic acid, ethyl ester	ND		26.51 ± 1.2	0.28 ± 0.1	Ester	C_19_H_38_O_2_	298.51
Ethyl 15-methyl-hexadecanoate	ND		26.57 ± 0.1	8.1 ± 0.4	Ester	C_19_H_38_O_2_	298.51
Z,Z-9,12-Octadecadienoic acid	26.90 ± 2.1	13.48 ± 0.4	27.05 ± 0.7	9.85 ± 0.2	Acid	C_18_H_32_O_2_	280.24
Oleic acid	26.95 ± 0.6	6.38 ± 0.8	ND		Acid	C_18_H_34_O_2_	282.47
9,17-Octadecadienal, (Z)	26.95 ± 1.5	6.78 ± 0.6	ND		Ketones	C_18_H_32_O	264.45
cis-13-Octadecenoic acid	27.15 ± 0.2	21.48 ± 1.1	27.15 ± 2.3	23.48 ± 0.4	Acids	C_18_H_34_O_2_	282.47
cis -Vaccenic acid	ND		27.21 ± 1.5	5.49 ± 0.4	Acids	C_18_H_34_O_2_	282.47
Linoleic acid ethyl ester	27.21 ± 1.2	11.46 ± 0.2	27.32 ± 1.7	12.87 ± 0.1	Esters	C_20_H_36_O_2_	308.51
Ethyl Oleate	27.21 ± 1.4	5.65 ± 0.3	27.25 ± 1.3	17.83 ± 0.3	Esters	C_20_H_38_O_2_	310.52
Beta-Sitosterol	27.98 ± 2.6	0.09 ± 0.2	ND		Alcohol	C_29_H_50_O	414.72
Ergosta-4,7,22-trien-3.beta.-ol	28.21 ± 0.7	2.15 ± 0.9	ND		Alcohol	C_28_H_44_O	396.34
Ergosterol	31.28 ± 1.7	5.33 ± 0.1	29.46 ± 0.5	4.3 ± 0.4	Alcohol	C_28_H_44_O	396.66

* Not detected, RT = Retention time

### Acute toxicological evaluation

The survival rate in the assay was 100%. In the acute toxicity study, methanolic extracts doses under 5000 mg/kg bw and spore doses under 1 × 10^9^ spores/mL did not cause any mortality or signs of toxicity in female mice over the observed period. No obvious clinical signs, including hair loss, scabbing, soft or mucoid feces, and lowered defecation, were noted in the study animals. All of the experimental animals showed normal behavior 14 days after administration, which demonstrates that the metabolites produced by the studied species as well as direct administration of live cells are safe for mice. Furthermore, the administration of metabolites or live cells did not cause any inflammatory or enterotoxigenic effects that could have induced gastroenteritis, e.g., diarrhea. On the other hand, it was not possible to calculate the mean lethal dose (LD_50_) because the administered doses did not cause death in any of the mice. In other words, the LD_50_ test results concerning methanolic extracts and spore suspensions mean that the metabolites and *Trichoderma* species do not cause any lethal effects in mice at doses below 5000 mg/kg bw and 1 × 10^9^ spores/mL, respectively.

### Effect of methanolic extracts and spore suspensions on body weight of mice

**Fig 4:**
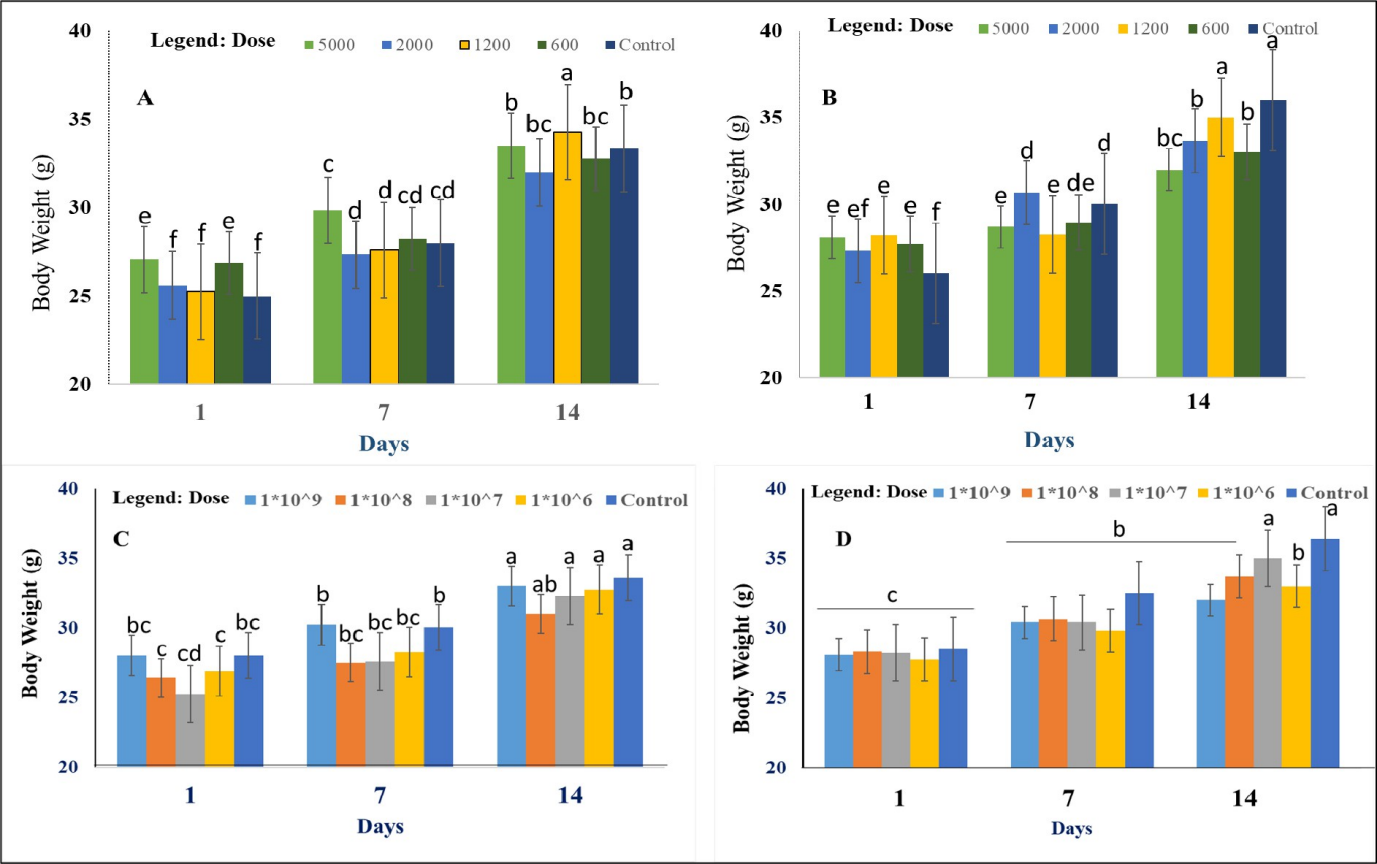
Changes in the body weights of mice administered with crude extracts and spore suspensions from *Trichoderma* species. (**A** and **C**) *T*. *asperellum* AU131 and (**B** and **D**) *T*. *longibrachiatum* AU158. (**A** and **B**) Mice that were administered with methanolic crude extracts and (**C** and **D**) mice that were administered with spore suspensions. Different alphabets depicted in superscript indicate mean treatments that are significantly different according to Tukey’s HSD posthoc test at p ≤ 0.05, each value is an average of 5 replicate samples ± standard error. Each point represents the mean value ± SD (n = 5).

Animals in the treatment group, i.e., which were administered with methanolic extracts or live cells, did not show any apparent changes in body weight, with the values similar to what was observed in the control group, which were administered with a 0.9% NaCl solution. The results illustrated in Fig 4A–4D demonstrate that mice in both the treatment and control groups showed a gradual increase in body weight over the study period. The results presented in this study showed that the oral administration of either methanolic extracts (Fig 4A and 4B) or cellular suspensions Fig 4C and 4D did not cause any significant (p > 0.05) changes in the weight of mice. Furthermore, the body weights of animals in the treatment group did not noticeably differ from the body weights of animals in the control group. This suggests that the methanolic extracts and spores of the two tested *Trichoderma* species neither support weight loss nor stimulate weight gain.

## Discussion

The present study investigated the *in vitro* bioassay and acute toxicity of these MBCAs through short-term assays in animal models to ensure their safety in coffee farm applications. Most of the species involved in *Trichoderma* infections are considered as opportunistic pathogens [46], with the virulence factors including mycelial growth at 37°C and neutral pH, hemolytic activity and toxicity to mammalian cells [13, 14], along with resistance to antifungal compounds [47]. Acute toxicity refers to the toxic effects of substances that result either from a single or multiple doses in a short period of time (usually less than 24 h) and can be described within 14 days [42]. The results of the present study showed that neither *T*. *asperellum* AU131 nor *T*. *longibrachiatum* AU158 grew on minimal media at 37°C. However, both species were able to grow at physiological pH, which agrees with the reports of Antal et al. [13] and Kredics et al. [14]. More specifically, Antal et al. [13] reported that *T*. *longibrachiatum* strains showed optimum growth at 30°C on both minimal and yeast extract agar media, while all of the strains were also able to grow at 40°C. Mikkola et al. [48] reported that toxic *T*. *longibrachiatum* strains grew on malt extract agar (MEA) at 22°C and 37°C. Hence, the concerns and risks associated with biopesticides can differ from one species to another, most likely requiring a case-by-case approach in associated risk assessment.

*Trichoderma* species are known to produce a wide range of bioactive secondary metabolites that are known to have antifungal, antibacterial, and toxic properties to control a wide range of phytopathogens, such as *Fusarium* species, *Botrytis cinerea*, *Pythium* species, *Rhizoctonia solani*, *Sclerotinia sclerotiorum*, and *Ustilago maydis* [8, 21, 37]. In the present study, the results of the antifungal activity of methanolic extracts obtained from both *T*. *asperellum* AU131 and *T*. *longibrachiatum* AU158 showed that the extracted secondary metabolites inhibited the growth of *F*. *xylarioides* in the seeded agar and agar diffusion assays, as shown in Fig 2A and 2B. Metabolic extracts from both bioagents have antifungal activity against *F*. *xylarioides*, however, the extract from *T*. *longibrachiatum* AU158 was more effective, as it was able to inhibit at the minimum concentration (10 mg/mL) in seeded agar assay. Many species of *Trichoderma*, such as *T*. *harzianum*, *T*. *viride*, *T*. *hamatum*, *T*. *atroviride*, and *T*. *virens*, have been reported to be effective in control of a wide range of soil borne plant pathogens [49, 50]. However, the biocontrol efficacy varies with *Trichoderma* species and target plant diseases [51]. A significant advancement from the present study is the finding that the SSF and methanolic extracts of the AU131 and AU158 strains provided a significant inhibitory effect on *F*. *xylarioides*, a causative agent of coffee wilt disease. Our findings suggest that both strains can be considered as a biocontrol agent in the effort of using alternative approaches to control coffee wilt.

Analysis of the GC-MS chromatograms showed that both MBCAs produced large quantities of VOCs that were identified as alcohols, acids, esters, sesquiterpenes and ketones. This wide range of VOCs emitted by both *Trichoderma* species is in line with the diverse VOCs produced by *Trichoderma* species reported in previous studies [25, 37]. The volatile secondary metabolites identified from methanolic extracts of the two investigated *Trichoderma* species (*T*. *asperellum* AU131 and *T*. *longibrachiatum* AU158) also agreed with what has previously been reported in the literature [32, 33, 37, 52]. The fungal production of VOCs is a dynamic process in that it is directly affected by both genetic and environmental factors, which include community composition, substrate, temperature, moisture level, and pH [53–56]. The identified metabolites play important roles in mycoparasitic interactions as well as induced systemic resistance (ISR) in plants via the upregulation of jasmonic acid and salicylic acid synthesis [57]. For instance, 2,4-bis(1,1-dimethylethyl)-phenol (2,4-DTBP) was found to be effective against the agriculturally important root-rot fungus; *Fusarium oxysporum* based on the inhibition of spore germination and hyphal growth [58]. During fungal spore germination, 2,4-DTBP completely inhibited germination by preventing the emergence of a normal germ tube, eventually leading to abnormal branching and swelling of the hyphae [58].

As GC-MS is the first step towards profiling the metabolites present in methanolic extracts, the present study showed presence of five major VOCs from both BCAs, *viz*., cis-13-Octadecenoic acid, 2,4-bis(1,1 dimethylethyl)-Phenol, *n*-hexadecanoic acid, ethyl oleate and hexadecanoic acid ethyl ester (Fig 3A and 3B). Most of the metabolites identified in this study are widely used as antimicrobials, food additives, cancer drugs, herbicides and pesticides. Specifically, cis-13-Octadecenoic acid and *n*-hexadecanoic acid are reported to have anti-inflammatory, cancer preventive and hepatoprotective properties [59]. The other identified linoleic acid ester and Z,Z-9-12-Octadecenoic acid also have anti-inflammatory, antiandrogenic, and anemiagenic properties [60]. Moreover, two of the identified volatile compounds–tetradecanoic acid and *n*-hexadecanoic acid methyl ester–are antioxidants [61]. Since there are no complete toxicity reports for all the secondary metabolites from *Trichoderma* species, we tested their toxicological levels using animal models (mice). We used methanolic extracts to assess the toxicological risks associated with a particular MBCA rather than using purified commercial metabolites as per the guidelines of the OECD.

Methanolic extracts and spore suspensions were first administered by oral gavage, since this is the most common route of human exposure. Thus, the acute toxicity assays were performed to monitor the harmful effects of an MBCAs to the organism following single or short-term exposure [62]. The performed experiments mainly evaluated mortality, changes in behavior, body weight, and other characteristics relative to the overall well-being of mice. In the present study, the *in vivo* toxicity evaluation of methanolic extracts and spore suspensions of *Trichoderma* species did not reveal any mortality among the Swiss albino mice; this suggests that the extracts and spore suspensions are not deadly to mammals. A report by the European Food Safety Authority EFSA [63] indicated that *T*. *harzianum* Rifai strain T-22 and *T*. *asperellum* T25 did not result in adverse effects following the oral, intratracheal, subcutaneous and intravenous administration of doses ranging from 6.4 × 10^6^ to 1.5 × 10^7^ colony forming unit (cfu)/animal. Moreover, the report indicated complete clearance of the spore suspension within two days of oral administration, a dramatic decrease in levels in the lungs by day 21 post-intratracheal administration, and a marked decrease in the number of cfu from the colonized organs following intravenous administration. No signs of pathogenicity were observed for any of the routes of exposure, which is in agreement with the results of the present study.

However, methanolic extracts of various *T*. *longibrachiatum* isolates have been reported to contain heat-resistant substances that inhibit the motility of boar spermatozoa and quench the mitochondrial transmembrane potential (ΔΨm) of sperm cells at low concentrations [64]. Several clinical isolates originally identified based on their morphological characters were recently re-identified by sequence-based molecular techniques as *T*. *longibrachiatum*, which provided the most frequently occurring clinical etiological agent within the genus *Trichoderma* [65, 66]. Thus, the biotechnological and agricultural application of *T*. *longibrachiatum* strains should be carefully monitored to minimize possible health risks.

Methanolic extracts from *T*. *asperellum* AU131 showed no toxicity up to concentrations of 5000 mg/kg bw. Moreover, acute oral toxicity studies conducted with both *T*. *asperellum* AU131 and *T*. *longibrachiatum* AU158 cellular suspensions did not result in treatment-related adverse effects across doses ranging from 1 × 10^6^ to 1 × 10^9^ spores/mL. This was supported by previous observations that the oral administration of methanolic extracts and cellular suspensions representing various fungal BCAs demonstrated very low toxicity in rodents and ruminants [28, 67]. Moreover, a report from the USA Environmental Protection Agency [27] demonstrated that *T*. *asperellum* strain ICC 012 is non-toxic to rats at a dose of 2000 mg/kg bw and 4.2 × 10^9^ cfu/g. The product formulated from this species contains 99.9% *w/w T*. *asperellum* strain ICC 012 as the active ingredient, with a minimum and nominal content of 1 × 10^9^ and 2.5 × 10^9^ cfu/g dry weight), respectively. The report clearly stated that exposure to this species does not cause any acute, sub-chronic, chronic, immune, endocrine, or non-dietary issues.

On the other hand, the oral median lethal dose (LD_50_) could not be calculated since the administered doses did not cause any death to the mice. These results suggest that the lethal doses for both *T*. *asperellum* AU131 and *T*. *longibrachiatum* AU158 methanolic extracts exceed 5000 mg/kg bw, which represents the highest reference dose [17]. In this regard, methanolic extracts may be considered safe at the tested levels, i.e., 5000 mg/kg bw and below. Changes in body weight is an important index for evaluating the toxicity of bioformulated products [68]. In the present study, both the treatment and control groups demonstrated a gradual increase in mean body weight. However, the difference in weight gain between the control and treatment groups was statistically insignificant (*p* ≥ 0.05).

Moreover, the study evaluate the acute toxicity of *T*. *asperellum* AU131 and *T*. *longibrachiatum* AU158 using Swiss albino mice and GC-MS to identify volatile metabolites of methanolic extracts. However, non-volatile metabolites produced by *Trichoderma* species and showing antimicrobial activity such as gliotoxin, peptaibols, gliovirin, polyketides, and pyrones [69–71] were not addressed in this study since they are not detected by GC-MS. Thus, further study is in progress to quantify and assess their toxicity related risks by using untargeted liquid chromatography–high resolution mass spectrometry (LC–HRMS).

## Conclusion

In conclusion, methanolic extracts and spore suspensions from *T*. *asperellum* AU131 and *T*. *longibrachiatum* AU158 were neither toxic nor pathogenic to Swiss albino mice across the tested range of concentrations. A GC-MS analysis of the extracts from both bioagents revealed the presence of 23 VOCs that were classified as alcohols, acids, sesquiterpenes, ketones and aromatic compounds. Both *Trichoderma* species showed optimum growth at 28°C on minimal and yeast extract agar media, with neither species growing at 37°C. In addition, methanolic extracts from *T*. *longibrachiatum* showed the highest inhibition activity and was active even at a low concentration (10μg/mL). In general, the study provides good insight to use these mBCAs as antagonists in biocontrol of CWD, *F*. *xylarioides*.
